# Human Immunodeficiency Virus (HIV)-Negative and Human Herpes Virus-8 (HHV-8)-Positive Primary Effusion Lymphoma: A Case Report and Review of the Literature

**DOI:** 10.4274/tjh.53215

**Published:** 2013-03-05

**Authors:** Sonay Güven Karataş, Reyhan Bayrak, Özlem Şahin Balçık, Kadir Serkan Yalçın, Esra Atıcı, Ümit Akyıldız, Ali Koşar

**Affiliations:** 1 Turgut Özal University, School of Medicine, Department of Internal Medicine, Ankara, Turkey; 2 Turgut Özal University, School of Medicine, Department of Pathology, Ankara, Turkey; 3 Turgut Özal University, School of Medicine, Department of Hematology, Ankara, Turkey

**Keywords:** Differential diagnosis, Human herpes virus-8, Human immunodeficiency virus (HIV), Pleural effusion, Primary effusion lymphoma

## Abstract

Primary effusion lymphoma (PEL) is a rare type of non-Hodgkin lymphoma that presents with serosal effusion in body cavities, without obvious tumor masses. Although PEL occurs in immunocompromised patients that are human immunodeficiency virus (HIV) positive, it also occurs in immunocompetent human herpes virus-8 (HHV-8)-positive patients. Herein we present an immunocompetent, HIV-negative, CD-20-negative, HHV-8-positive patient with pleural effusion that was diagnosed as PEL. The CHOP protocol and talc pleurodesis were administered. HHV-8 plays a causative role in PEL and is important for differentiating PEL from other types of lymphoma. As such, in addition to pleurodesis antiviral treatment should be considered for optimal treatment outcome.

**Conflict of interest:**None declared.

## Abstract (tr)

Primer efüzyon lenfoması (PEL) vücut boşluklarındaki seröz zarlarda tümör kitlesi olmaksızın, sadece serozal effüzyonla karakterize, nadir görülen bir hodgkin dışı lenfomadır. PEL immünsupresif ve özellikle HIV pozitif olgularda görülmekle birlikte Human herpes virus-8 (HHV-8) pozitif immün sistemi sağlam olgularda da tespit edilmektedir. Bu olguda immünkompetan, HIV negatif, plevral effüzyonu olan HHV-8 pozitif, primer effüzyon lenfomalı erkek hasta sunulacaktır. HHV-8 pozitif CD-20 negatif PEL olgumuzda tedavide CHOP protokolü ve talk plörodezis uygulandı. HHV-8 pozitifliği PEL tanı ve ayırıcı tanısında önemli role sahiptir. Bu yüzden, PEL tedavisinde plörodezise ek olarak antiviral tedavinin uygulanmasıyla başarılı sonuçlar elde edilebileceği düşünülmektedir.

## INTRODUCTION

Primary effusion lymphoma (PEL) is a rare type of non-Hodgkin lymphoma that presents with pleural, pericardial, or peritoneal serosal effusion in body cavities, without detectable tumor masses or lymphadenopathies. PEL usually occurs in 1 body cavity; however, some cases involving more than 1 cavity have been reported [1]. PEL primarily affects human immunodeficiency virus (HIV)-positive patients and immunocompromised patients with malignancy, cirrhosis, or solid organ transplantation; it rarely occurs in immunocompetent patients. Immunocompetent patients diagnosed as PEL are usually more than 60 years old, infected with human herpes virus-8 (HHV-8), and in some cases infected with Epstein Barr virus (EBV) [1,2,3,4,5,6]. PEL is considered a non-Hodgkin lymphoma (NHL) associated with HHV-8, HIV, and EBV [6]. Herein we present an HIV-negative, EBV-negative, HHV-8-positive immunocompetent male patient with PEL. Flow cytometry was not performed in our hospital because we do not have enough medical equipment for this technic.

## CASE REPORT

**Clinical Summary **

**Figure 1:**
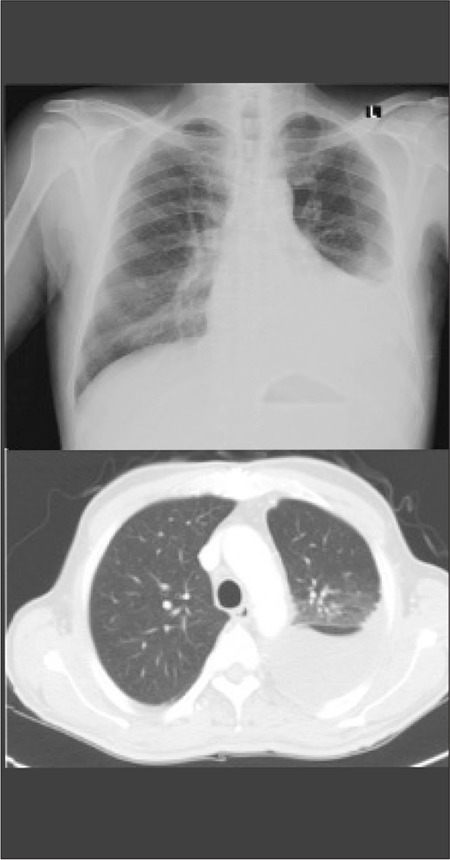
Chest X-ray and chest CT show a massive, left-sided pleural effusion.

**Figure 2:**
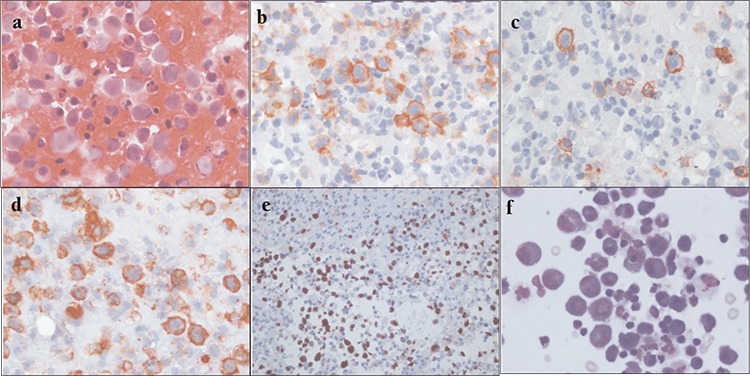
A-F: Cytomorphology and immunophenotype of the PEL cells. Large, atypical lymphoid cells exhibit rounded nuclei, coarsely granular chromatin, and a moderate amount of cytoplasm. Apoptotic bodies and necrotic debris are seen throughout the smears. Many of the smudged, small nuclei represent pyknosis (individual cell necrosis-not small lymphocytes (H&E, 1000×) (A-F a. Mitotic figures were abundant (A). Expression of CD30 (B), CD138 (C), epithelial membrane antigen (EMA) (D), and HHV-8 (E) in tumor cells (immunoperoxidase, 1000×). Cytomorphology of the PEL cells in the smears of ascites specimen (F).

A 72-year-old male was admitted to hospital due to a 4-week history of anorexia, fatigue, shortness of breath, and non-productive cough. He has had hyperlipidemia and type-2 diabetes mellitus for 20 years, and underwent coronary artery bypass grafting surgery in 1990. Breath sounds were absent on the left side. Laboratory findings are shown in the Table. Chest X-ray and chest computed tomography (CT) showed a massive, left-sided pleural effusion (Figure 1). Peripheral lymphadenopathy (LAP) and organomegaly were not observed. Bone marrow biopsy was not performed because the patient did not want the procedure. Positron emission tomography-computed tomography (PET-CT) showed that there was no signicifiant findings. Echocardiography showed 40% ejection fractions, but no pericardial effusion. Due to the pleural effusion thoracentesis (continuous drainage of the left-sided pleural effusion) and talc pleurodesis were performed. The pleural fluid was hemorrhagic and biochemical examination showed that it was exudate (lactate dehydrogenase [LDH]: 2386 U/L; albumin: 2.6 g/dL). Cytomorphological and immunocytochemical analysis of the pleural fluid showed high grade malignant lymphoma and the patient was diagnosed as HHV-8-positive PEL (Figure 2A-E).

The CHOP chemotherapy protocol (cyclophosphamide 750 mg/m^2^, adriamycin 50 mg/m^2^, and vincristine 1.4 mg/m^2^ on d 1, and prednisolone 100 mg on d 1-5; the cycle is repeated every 21 d) was initiated 1 month post diagnosis. After the first cycle cerebral vascular insufficiency was observed and therefore the protocol was discontinued. During follow-up the patient’s pleural effusion did not recur, but progressive abdominal swelling was observed 4 months post diagnosis. Abdominal ultrasonography showed significant ascites and chest X-ray showed the absence of pleural effusion. Paracentesis was performed because of the ascites. Cytomorphological and immunocytochemical analysis of the ascites showed similar findings as for the pleural fluid. HHV-8-positive PEL was observed in ascites as well (Figure 2F). 2 months after paracentesis the patient returned to the hospital with recurrent ascites and paracentesis was performed again. Right-sided pleural effusion was observed 3 months after the last paracentesis. Thoracentesis and talc pleurodesis were performed due to pleural effusion. The right-sided pleural fluid showed similar findings as that for the left-sided pleural fluid examined earlier. The patient was still alive 29 months after diagnosis.

**Cytomorphological and Immunocytochemical Analysis**

Figure 2A-F shows the cytomorphology and immunophenotype of the PEL cells. Smears or cytospins were prepared from the pleural fluid and ascites specimens, and were stained with hematoxylin and eosin (H&E), Giemsa, and Papanicolaou (Pap) methods. Cell blocks were fixed in 4% buffered formaldehyde and stained with H&E. Immunophenotypic profiles were determined according to standard immunoperoxidase methods, using paraffin sections from the cell block. The primary antibodies used for immunocytochemistry were as follows: CD2 (clone AB75, NeoMarkers); CD3 (clone SP7, NeoMarkers); CD4 (clone 4B12, DAKO); CD5 (clone CD5/54/F6, DAKO); CD7 (clone 272, NeoMarkers); CD8 (clone C8/144B, DAKO); CD19 (clone LE-CD19, DAKO); CD20 (clone L26, NeoMarkers); CD30 (clone Ber-H2, DAKO); CD43 (clone DF-T1, NeoMarkers); CD45 (Clone 2B11 + PD7/26, DAKO); CD138 (clone M115, DAKO); ALK-1 (clone ALK01, NeoMarkers); EMA (clone E29, DAKO); EBV/LMP (clone CS. 1-4, NeoMarkers); HHV-8/LANA (clone 13B10, Novacastra). Tumor cells did not express CD2, CD3, CD4, CD5, CD7, CD8, CD19, CD20, CD45, anaplastic lymphoma kinase (ALK), or EBV/LMP. 

## DISCUSSION

PEL is characterized by proliferation of malignant lymphocytes in the major body cavities (without detectable solid masses), large-cell, immunoblastic, plasmablastic, or anaplastic cytomorphology, B-cell lymphoma that often has an indeterminate immunophenotype based on non-molecular methods, malignancy that usually develops in immunocompromised patients (especially those that are HIV-positive), and the universal presence of HHV-8 [7]. 

Although PEL is B-cell clonal in origin, it exhibits a non-B, non-T null phenotype. The neoplastic cells in PEL lack surface expression of B-cell markers, such as CD19, CD20, CD79a, and immunoglobulin, they express plasma cell-related markers, such as CD30, CD38, CD45, and CD138, but they also exhibit clonal rearrangement and somatic hypermutation of immunoglobulin genes, suggesting that they originate from post-germinal center B-cells [1,5,6,7]. Cytologically, PEL is a large cell lymphoma with both morphologic features of immunoblastic and anaplastic large cell lymphomas; therefore, cytomorphological differentiation of PEL from other lymphomas involving the body cavities may be difficult. The major differential diagnoses include anaplastic large cell lymphoma (ALCL), diffuse large B-cell lymphoma (DLBCL), Burkitt’s lymphoma, and pyothorax-associated lymphoma (PAL). 

ALCL is a T-cell/null-cell lymphoma that typically involves lymph nodes or extra nodal sites, such as skin, soft tissue, and bone. ALCL presenting with pleural effusion has rarely been reported [8]. Also ALCL and PEL may overlap, both morphologically and immunophenotypically. The distinction between these 2 entities is important because ALCL has a much better prognosis. Morphologically, they are both high-grade lymphomas composed of large cells with pleomorphic nuclei and prominent, single-to-multiple nucleoli. Immunohistochemically, both entities usually stain positively for CD45, CD30, and EMA. Unlike PEL, ALCL usually expresses some T-cell markers and ALK-1; however, rare PEL cases express T-cell markers [9,10]. In addition, approximately 20%-40% of adult ALCL patients are negative for ALK-1 [11]; therefore, the importance of testing for the presence of HHV-8 in lymphoma cells cannot be overemphasized, because its detection is regarded as a sine qua non for the diagnosis of PEL, in contrast to ALCL. Burkitt’s lymphoma may present as a serous effusion, but is usually differentiated from PEL by its distinct morphologic features (i.e. medium-sized cells with round nuclei, prominent nucleoli, and basophilic vacuolated cytoplasm). Immunophenotyping usually shows a B-cell lineage in Burkitt’s lymphoma, as compared to the null-phenotype of PEL. Positive staining for CD10, Bcl-6, and c-my, and the absence of HHV-8 are also helpful findings in Burkitt’s lymphoma. DLBCL, including the immunoblastic variant, also overlaps with PEL. Although very rare cases of primary body cavity DLBCL have been reported [12], the majority of cases are associated with contiguous or disseminated disease at presentation. The immunophenotype and HHV-8 positivity are helpful in distinguishing these 2 entities, because DLBCL is HHV-8-negative and expresses B-cell surface markers. 

PAL is a pleural, EBV-associated NHL that develops following longstanding chronic pleural inflammation. It is not associated with systemic immunodeficiency, but is linked to local immunosuppression and chronic stimulation, inducing clonal proliferation of cells latently infected with EBV [13]. Unlike PEL, extensive local solid masses are also observed in PAL. Cytologically, PAL may be indistinguishable from PEL; as such, immunophenotyping and HHV-8 testing are essential for the differential diagnosis. PAL patients are HHV-8-negative and express B-cell markers. Furthermore, EBV detection, although important in the diagnosis of PAL also it could be seen in PEL, because of this reason the detection of EBV is not specific disorder for PAL [14].

PEL usually occurs in HIV-positive patients. HIV-negative PEL patients are in a immunodeficient state because of advanced age or some other conditions, including solid organ transplantation, cirrhosis, and malignancy [1,2,3,4,5,6,15]. The presented patient did not have any underlying immunodeficiency conditions, except for advanced age. A search for all series and case reports of HIV-negative and HHV-8-positive PEL cases published in the English-language literature as late as June 2011 was conducted. A review of the literature by Yiakoumis et al. reported only 20 HIV-negative PEL cases, as well as 2 PEL cases that were HIV-negative and HHV-8-positive. %83,6 of the patients were male and the mean age of all patients was 62 years. Some of the patients had associated Kaposi sarcoma (KS) or multicentric Castleman’s disease (MCD) [15]. The CHOP and modified CHOP protocols were administered to 11of the patients; only 4 of these patients survived more than 1 year [16]. In addition to these cases, we found reports on another 6 HIV-negative PEL cases [1,15,17]. 

CHOP combination chemotherapy regimens are most commonly used as first-line therapy for PEL, which usually result in survival of only a few months-prognosis is very poor. In a retrospective study median survival was 6.2 months and the 1-year overall survival rate was 39.3% [18]. HAART (highly-active antiretroviral therapy) is recommended in HIV-positive PEL patients and rituximab-CHOP chemotherapy is recommended for PEL patients that are HHV-8 negative and CD20 positive [6,18]. Tamara et al. reported good results with intracavitary cidofovir in HIV-negative, HHV-8-positive PEL patients [17]. Although PEL has a poor prognosis, use of CHOP chemotherapy, antiviral treatment after drainage of effusion, and intracavitary treatment after pleurodesis should be considered for improving survival. In the present case talc pleurodesis for pleural effusion was also performed and after this treatment any pleural effusion was not repeated on the same side. 

**Conflict of Interest Statement**

The authors of this paper have no conflicts of interest, including specific financial interests, relationships, and/ or affiliations relevant to the subject matter or materials included.

## Figures and Tables

**Table 1:**
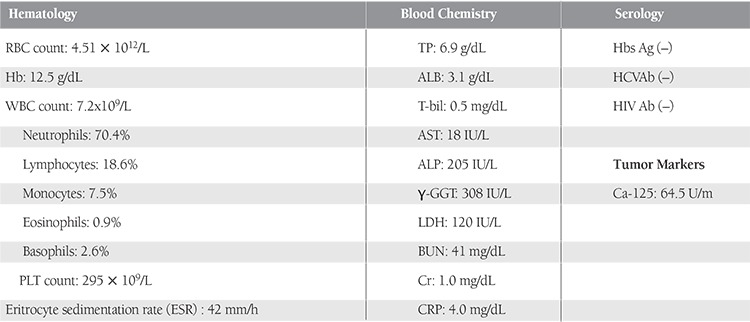
Laboratory findings.

## References

[1] Brimo F, Popradi G, Michel RP, Auger M (2009). Primary Effusion Lymphoma involving Three Body Cavities. Cytojournal.

[2] Ascoli V, Scalzo CC, Danese C, Vacca K, Pistilli A, Lo Coco F (1999). Human herpes virus-8 associated primary effusion lymphoma of the pleural cavity in HIV-negative elderly men. Eur Respir J.

[3] Ikebe T, Amemiya Y, Saburi M, Ando T, Kohno K, Ogata M, Hiramatsu K, Kadota J (2010). Rare Primary effusion Lymphoma Associated with HHV-8 in Japan. Intern Med.

[4] Ceran F, Aydin Y, Ozcakar L, Han U, Yildiz M (2009). Primary Effusion Lymphoma: An Untrivial Differential Diagnosis for Ascites. Yonsei Med J.

[5] Boulanger E, Hermine O, Fermand JP, Radford-Weiss I, Brousse N, Meignin V, Gessain A (2004). Human Herpesvirus 8(HHV-8) Associated Peritoneal Primary Effusion Lymphoma (PEL) in Two HIV-Negative Elderly Patients. Am J Hematol.

[6] Terasaki Y, Okumura H, Saito K, Sato Y, Yoshino T, Ichinohasama R, Ishida Y (2008). HHV-8/KSHV-Negative and CD20 Positive Primary Effusion Lymphoma Successfully Treated by Pleural drainage Followed by Chemoteraphy Containing Rituximab. Intern Med.

[7] Wakely PE Jr, Menezes G, Nuovo GJ (2002). Primary Effusion Lymphoma: Cytopathologic Diagnosis Using In Situ Molecular Genetic Analysis for Human Herpesvirus 8. Mod Pathol.

[8] Chan AC, Chan JK, Yan KW, Kwong YL (2003). Anaplastic large cell lymphoma presenting as a pleural effusion and mimicking primary effusion lymphoma. A report of 2 cases. Acta Cytol.

[9] Lechapt-Zalcman E, Challine D, Delfau-Larue MH, Haioun C, Desvaux D, Gaulard P (2001). Association of primary pleural effusion lymphoma of T-cell origin and human herpesvirus 8 in a human immunodeficiency virus-seronegative man. Arch Pathol Lab Med.

[10] Said JW, Shintaku IP, Asou H, deVos S, Baker J, Hanson G, Cesarman E, Nador R, Koeffler HP (1999). Herpesvirus 8 inclusions in primary effusion lymphoma: report of a unique case with T-cell phenotype. Arch Pathol Lab Med.

[11] Gascoyne RD, Aoun P, Wu D, Chhanabhai M, Skinnider BF, Greiner TC, Morris SW, Connors JM, Vose JM, Viswanatha DS, Coldman A, Weisenburger DD (1999). Prognostic significance of anaplastic lymphoma kinase (ALK) protein expression in adults with anaplastic large cell lymphoma. Blood.

[12] Fujisawa S, Tanioka F, Matsuoka T, Ozawa T (2005). CD5+ diffuse large B-cell lymphoma with c-myc/IgH rearrangement presenting as primary effusion lymphoma. Int J Hematol.

[13] Aozasa K (2006). Pyothorax-associated lymphoma. J Clin Exp Hematop.

[14] Hamoudi R, Diss TC, Oksenhendler E, Pan L, Carbone A, Ascoli V, Boshoff C, Isaacson P, Du MQ (2004). Distinct cellular origins of primary effusion lymphoma with and without EBV infection. Leuk Res.

[15] Brimo F, Michel RP, Khetani K, Auger M (2007). Effusion Lymphoma. A series of 4 cases and Review of the Literature With Emphasis on Cytomorphologic and Immunocytochemical Differential Diagnosis. Cancer.

[16] Yiakoumis X, Pangalis GA, Kyrtsonis MC, Vassilakopoulos TP, Kontopidou FN, Kalpadakis C, Korkolopoulou P, Levidou G, Androulaki A, Siakantaris MP, Sachanas S, Andreopoulos A (2010). Primary Effusion lymphoma in Two HIV-Negative Patients Successfully Treated with Pleurodesis as First-line Theraphy. Anticancer Res.

[17] Moyo TK, Richards KL, Damania B (2010). Use of Cidofovir for the Treatment of HIV- negative Human Herpes Virus-8 Associated Primary Effusion Lymphoma. Clin Adv Hematol Oncol.

[18] Boulanger E, Gerard L, Gabarre J, Molina JM, Rapp C, Abino JF, Cadranel J, Chevret S, Oksenhendler E (2005). Prognostic factors and outcome of human herpesvirus 8- associated primary effusion lymphoma in patients with AIDS. J Clin Oncol.

